# Al_x_CoCrFeNi High-Entropy Alloys Enable Simultaneous Electrical and Mechanical Robustness at Thermoelectric Interfaces

**DOI:** 10.3390/ma18153688

**Published:** 2025-08-06

**Authors:** Xiaoxia Zou, Wangjie Zhou, Xinxin Li, Yuzeng Gao, Jingyi Yu, Linglu Zeng, Guangteng Yang, Li Liu, Wei Ren, Yan Sun

**Affiliations:** 1 Engineering Research Center of Complex Track Processing Technology & Equipment, Ministry of Education, Xiangtan University, Xiangtan 411105, China; 2 School of Mechanical Engineering and Mechanics, Xiangtan University, Xiangtan 411105, China; 3 College of Semiconductors (College of Integrated Circuits), Hunan University, Changsha 410082, China

**Keywords:** skutterudites, high-entropy alloys, interface, interfacial resistivity, shear strength

## Abstract

The interface between high-performance thermoelectric materials and electrodes critically governs the conversion efficiency and long-term reliability of thermoelectric generators under high-temperature operation. Here, we propose Al_x_CoCrFeNi high-entropy alloys (HEA) as barrier layers to bond Cu-W electrodes with p-type skutterudite (p-SKD) thermoelectric materials. The HEA/p-SKD interface exhibited excellent chemical bonding with a stable and controllable reaction layer, forming a dense, defect-free (Fe,Ni,Co,Cr)Sb phase (thickness of ~2.5 μm) at the skutterudites side. The interfacial resistivity achieved a low value of 0.26 μΩ·cm^2^ and remained at 7.15 μΩ·cm^2^ after aging at 773 K for 16 days. Moreover, the interface demonstrated remarkable mechanical stability, with an initial shear strength of 88 MPa. After long-term aging for 16 days at 773 K, the shear strength retained 74 MPa (only 16% degradation), ranking among the highest reported for thermoelectric materials/metal joints. Remarkably, the joint maintained a shear strength of 29 MPa even after 100 continuous thermal cycles (623–773 K), highlighting its outstanding thermo-mechanical stability. These results validate the Al_x_CoCrFeNi high-entropy alloys as an ideal interfacial material for thermoelectric generators, enabling simultaneous optimization of electrical and mechanical performance in harsh environments.

## 1. Introduction

Thermoelectric generators (TEGs), which directly convert heat into electricity via the Seebeck effect, hold significant promise for various applications including deep-space exploration, automotive exhaust heat recovery, industrial waste heat harvesting, and wearable self-powering systems [1,2,3].

Considerable research has focused on enhancing the thermoelectric figure of merit (ZT), leading to the development of numerous high-performance thermoelectric material systems, including Mg_3_(Bi,Sb)_2_ [4], oxides [5], SnTe [6], skutterudites [7], half-Heusler compounds [8], and GeTe [9]. However, to achieve reliable TEG operation, these high-performance thermoelectric materials must be reliably bonded to metal electrodes to form high-performance thermoelectric devices. Consequently, the reliability of thermoelectric generators critically depends on the interfacial electrical contact stability and mechanical bonding strength between thermoelectric materials and metal electrodes [10,11,12,13,14,15,16]. The operational conditions for thermoelectric generators are exceptionally harsh conditions characterized by prolonged exposure to high temperatures on the hot side [17,18,19,20]. For instance, in deep-space exploration missions, the hot-side temperature of thermoelectric generators is subjected to 773 K, with continuous thermal contact durations potentially spanning several decades. In applications such as automotive exhaust heat recovery [21], where the heat source temperature fluctuates frequently and the mechanical environment is complex, thermoelectric generators are subjected to high-frequency thermal shocks and mechanical vibration loads. Therefore, thermoelectric generators must possess excellent stability under combined thermal–mechanical conditions.

The stability of thermoelectric generators is primarily threatened by the continuous microstructural evolution at their interfaces during thermal service. At the interface between thermoelectric materials and electrodes, progressive elemental diffusion during thermal operation leads to thickening of the interfacial reaction layer, resulting in increased parasitic electrical resistance and reduced conversion efficiency of the thermoelectric generators. To address this challenge, many studies have adopted the strategy of inserting a barrier layer between the thermoelectric material and electrode. This barrier layer serves dual critical functions: controlling elemental diffusion at the interface to mitigate the aging-induced increase in interfacial resistance and simultaneously enabling thermal expansion coefficient matching to enhance the shear strength between the thermoelectric material and electrode. The combined action of these effects leads to significant enhancement of the thermoelectric–electrode interface’s thermomechanical stability.

The optimization of interfacial properties typically requires tailored interfacial microstructure design. However, achieving both low interfacial resistance and high shear strength at the thermoelectric material/electrode interface using a single layer remains extremely challenging. Current interfacial layer materials for skutterudite/electrode joints primarily include pure metals (e.g., Ti, Nb) [22,23] and alloys (e.g., Ti-Al alloys [24], Ti-Al-Si alloys [25], Ni-Cr-Cu alloys [26], and Mo-Ti alloys [27]). Although certain studies have achieved effective control of interfacial elemental diffusion, thereby suppressing the accelerated growth of interfacial resistance during high-temperature aging, comprehensive mechanical characterization remains notably absent in many cases. The available literature indicates that the shear strengths of the thermoelectric/electrode interface are typically constrained to ~30 MPa, demonstrating concerning degradation to approximately 10 MPa post thermal aging.

High-entropy alloys (HEAs) possess exceptional characteristics such as sluggish diffusion effects and severe lattice distortion, which provide unique advantages in inhibiting atomic interdiffusion, along with excellent mechanical properties, thermal conductivity, and electrical conductivity [28,29,30]. These outstanding properties make HEAs promising interfacial materials between thermoelectric materials and electrodes for suppressing interfacial reaction layer growth during aging, reducing parasitic resistance, and improving mechanical performance simultaneously. In this study, we employed an Al_x_CoCrFeNi HEA as a barrier layer between p-type skutterudites (p-SKD) and Cu-W electrodes, achieving excellent physicochemical compatibility with skutterudites. The multiple constituent elements in Al_x_CoCrFeNi can chemically interact with the skutterudite layer to form complex intermetallic compounds, notably (Fe, Ni, Co, Cr)Sb. These intermetallic reaction layers exhibit several beneficial characteristics during thermal aging, including a slow growth rate, resistance to void formation and other diffusion-induced defects, and low interfacial electrical resistance. After prolonged aging at 773 K for 16 days, the interface demonstrated minimal layer growth while maintaining both low contact resistivity (7.15 μΩ·cm^2^) and ultra-high shear strength (74 MPa). Moreover, the joint retained good mechanical strength during continuous extreme thermal cycling tests. These results strongly support that our Al_x_CoCrFeNi HEA interlayer design can enable reliable operation of thermoelectric generators under complex thermo-mechanical conditions.

## 2. Materials and Methods

Al, Co, Cr, Fe, and Ni powders (45 μm, 99.95% purity) were precisely weighed according to the nominal compositions of Al_x_CoCrFeNi (specifically Al_0.3_CoCrFeNi and Al_1_CoCrFeNi). The elemental powders were loaded into a stainless steel jar within an argon-filled glove box and processed using a high-energy ball mill (SPEX 8000M) for 30 h. Milling durations under 30 min are insufficient to ensure adequate elemental interdiffusion, hindering the formation of the desired multi-component solid-solution phase of Al_x_CoCrFeNi high-entropy alloys (HEAs). A ball-to-powder weight ratio of 10:1 was maintained, with methanol added as a process control agent. The resulting Al_x_CoCrFeNi powder was then consolidated onto Cu_40_W_60_ electrode substrates via spark plasma sintering (SPS). Al_x_CoCrFeNi could fail to achieve full densification at low sintering temperatures and undergoes phase decomposition at excessively high temperatures. To strike a balance, SPS was performed at 900 °C for 5 min under a uniaxial pressure of 90 MPa in an argon atmosphere, followed by furnace cooling to ambient conditions. This process successfully yielded bilayer Al_x_CoCrFeNi/Cu–W joints. Finally, the p-SKD powder (La_0.8_Ti_0.1_Ga_0.1_Fe_3_CoSb_12_) was SPS-consolidated on the Al_x_CoCrFeNi layer, creating the complete p-SKD/Al_x_CoCrFeNi/Cu-W sandwich structure. The p-SKD layer was sintered at 650 °C for 10 min under 60 MPa, following identical conditions to those employed in our prior skutterudite studies [31,32]. This consistency ensures preservation of the p-SKD layer’s intrinsic thermoelectric properties. The primary fabrication methods used in this work—i.e., ball milling and spark plasma sintering (SPS)—are both widely employed in large-scale industrial production. Although the present experiments were conducted using laboratory-scale equipment, the overall processing route is straightforward and highly scalable. Therefore, the approach demonstrated in this work can be readily adapted to industrial-scale applications.

Phase identification of both powder precursors and consolidated bulk samples was performed using polycrystalline X-ray diffraction (XRD, Bruker D8 Advance). Microstructural characterization was conducted via scanning electron microscopy (SEM, JSM7610FPIus) equipped with energy-dispersive X-ray spectroscopy (EDS) for chemical composition analysis. Contact resistivity measurements were carried out using a custom-designed four-point probe system (equipped with a Keithley 2450 Source Measure Unit and 7510 Digital Multimeter). Mechanical shear testing was performed on a universal testing machine (AGX-V 50KN) with a 10 KN load cell at a constant crosshead speed of 0.2 mm/min. The thermal cycling tests of the joints were conducted in a Joule heating furnace (Particle Precision Instruments Co., Ltd., Changchun, China) with temperature cycling between 623 K and 773 K. The heating and cooling rates were maintained at 100 K/min throughout the thermal cycling process, with 5-second dwell times at both the upper (773 K) and lower (623 K) temperature limits. A total of 100 complete thermal cycles were performed.

## 3. Results and Discussion

**Figure 1:**
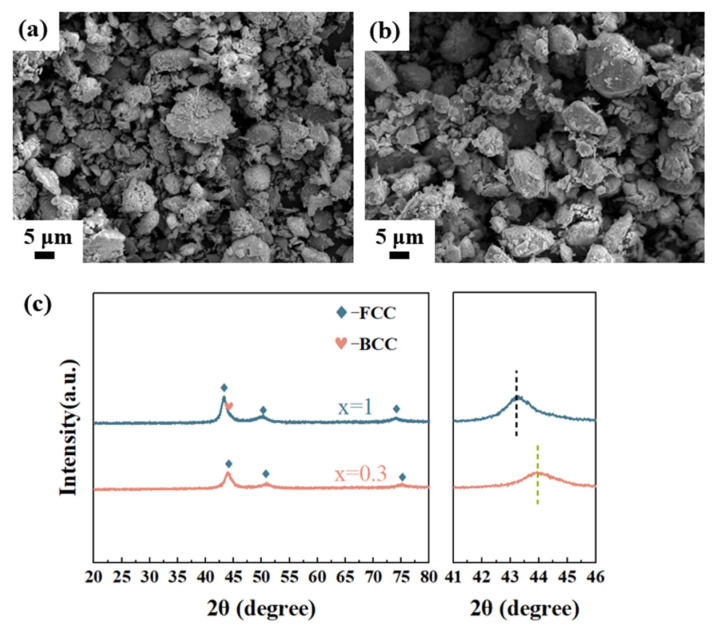
(**a**) SEM images of Al_0.3_CoCrFeNi and (**b**) Al_1_CoCrFeNi HEA powders, along with (**c**) their corresponding XRD patterns.

The Al_0.3_CoCrFeNi and Al_1_CoCrFeNi high-entropy alloy (HEA) powders were synthesized via mechanical alloying, followed by comprehensive characterization including SEM observation, EDS analysis, and XRD phase identification. As shown in Figure 1a,b, the as-milled powders primarily exhibit particle sizes of 5–10 μm, with nano-scale particles adhering to the powder surfaces. EDS measurements were performed at five randomly selected locations for each of the two high-entropy alloy powders. For all measured points, the relative deviation of Co, Cr, Fe, and Ni from the average composition was less than 10%, while that of Al was within 20%. The actual average compositions of the two high-entropy alloy powders are summarized in Table 1. EDS results (Table 1) also confirm that the actual compositions closely match the nominal compositions. The XRD patterns in Figure 1c demonstrate that the Al_0.3_CoCrFeNi alloy crystallizes in a single-phase face-centered cubic (FCC) structure, with all constituent elements achieving complete solid solubility, in agreement with prior reports from the literature [33,34,35]. Close examination of the diffraction peaks between 41° and 46° demonstrates distinct phase characteristics; while the Al_0.3_CoCrFeNi alloy shows symmetric FCC peaks, the Al_1_CoCrFeNi alloy exhibits asymmetric peak broadening toward the higher-angle side. This right-shoulder broadening indicates the coexistence of a body-centered cubic (BCC) phase with the primary FCC phase in Al_1_CoCrFeNi, as confirmed by reference to the standard peak positions reported in the literature [35]. The phase evolution suggests that increasing Al content promotes BCC phase formation, resulting in a dual-phase (FCC+BCC) structure in Al_1_CoCrFeNi. Due to the superior mechanical properties associated with FCC-structured alloys, such as lower yield strength, they are expected to accommodate interfacial stress through plastic deformation at the joint interface. Consequently, the p-SKD/Al_0.3_CoCrFeNi/Cu–W joint is anticipated to exhibit enhanced mechanical performance, as will be demonstrated in the following sections.

**Figure 2:**
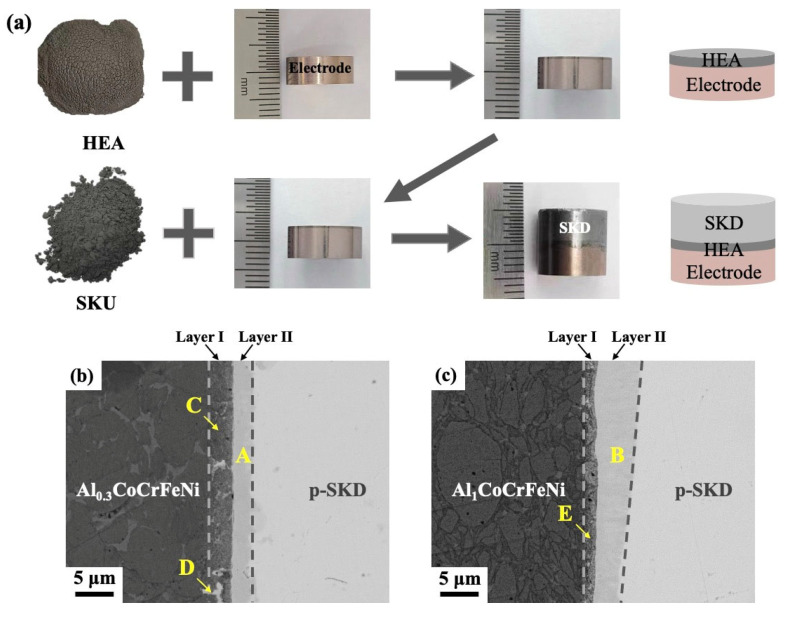
(**a**) Schematic illustration of the sequentially hot-pressed Cu-W/Al_x_CoCrFeNi/p-SKD joint and cross-sectional SEM images of the diffusion-bonded interfaces between p-SKD and (**b**) Al_0.3_CoCrFeNi or (**c**) Al_1_CoCrFeNi high-entropy alloys.

Al_x_CoCrFeNi (x = 0.3, 1) HEAs were utilized as barrier layers to bond Cu-W electrodes with skutterudite thermoelectric materials via sequential powder hot-pressing, where a 250 μm thick HEA layer was first sintered onto the Cu-W substrate followed by skutterudite consolidation, resulting in fully dense joints (Figure 2a). Microstructural characterization revealed well-bonded interfaces without voids or cracks for both Al_0.3_CoCrFeNi and Al_1_CoCrFeNi (Figure 2b,c), with distinct bilayer reaction zones consisting of a HEA-side layer (Layer I) and skutterudite-side layer (Layer II). The reaction layers showed different thicknesses depending on the HEA composition. On the skutterudite side, Al_1_CoCrFeNi formed a 4.3 μm layer, while Al_0.3_CoCrFeNi produced a thinner 2.5 μm layer. The HEA-side layers showed the opposite trend, being thicker for Al_0.3_CoCrFeNi than Al_1_CoCrFeNi. The two high-entropy alloys exhibit different diffusion-blocking efficiencies, which is likely attributable to their distinct crystalline structures. EDS results (Table 2) show both HEAs formed skutterudite-side layers with equal atomic ratios of Sb and (Fe+Cr+Co+Ni), confirming (Fe,Ni,Co,Cr)Sb phase formation. On the HEA side, the layers contained a dark gray matrix with light-gray particles. EDS results showed non-stoichiometric compositions due to the fine-scale mixing of two different phases, (Fe,Ni,Co,Cr)Sb and HEA phases. The measured compositions represent averaged values from both phases because the EDS probe size (~1 μm) exceeds individual phase dimensions.

**Figure 3:**
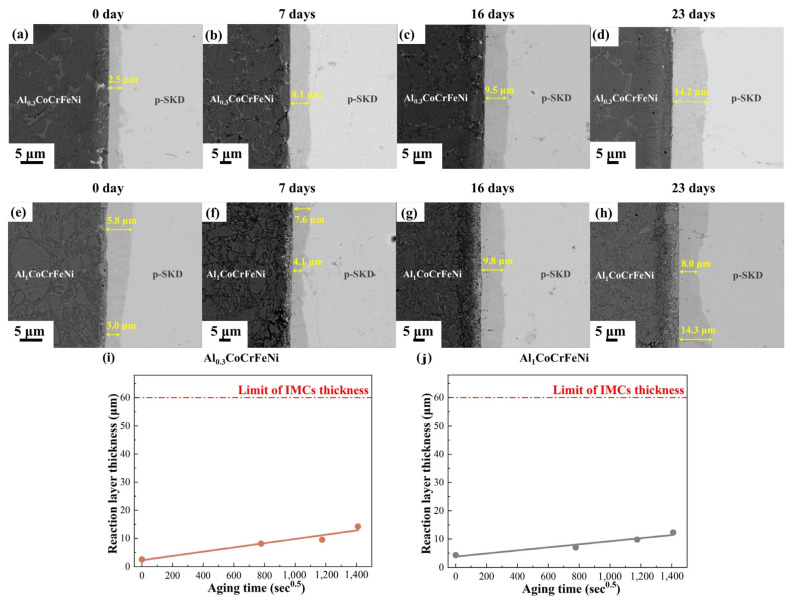
Microstructural evolution of Al_x_CoCrFeNi/p-SKD interfaces during aging treatment: (**a**–**d**) x = 0.3; (**e**–**h**) x = 1; (**i**,**j**) kinetic analysis of interfacial reactions.

To further evaluate the long-term service stability of the high-entropy alloy interface under high-temperature conditions, long-duration aging tests were conducted on the skutterudites/Cu-W joints at 773 K. As shown in Figure 3, with increasing aging time, the interfacial reaction layers formed by both high-entropy alloys exhibited no significant morphological contrast changes. Only a growth in the thickness of the reaction layers was observed, with no new phase formation. Phase analysis of the interfacial reaction layers after 500 °C/23-day aging was performed using XRD with a layer-by-layer removal technique. When performing layer-by-layer removal starting from either the p-SKD side or the high-entropy alloy side, the p-SKD or high-entropy alloy layer must be completely removed using abrasive paper until the reaction zone is fully exposed for XRD phase analysis. It should be noted that due to the extreme thinness of the interfacial reaction layer (a few micrometers), it is challenging to ensure perfect parallelism between the grinding surface and the reaction layer during polishing. This may result in XRD signals containing contributions not only from the reaction layer but also from residual p-SKD or high-entropy alloy material.

**Figure 4:**
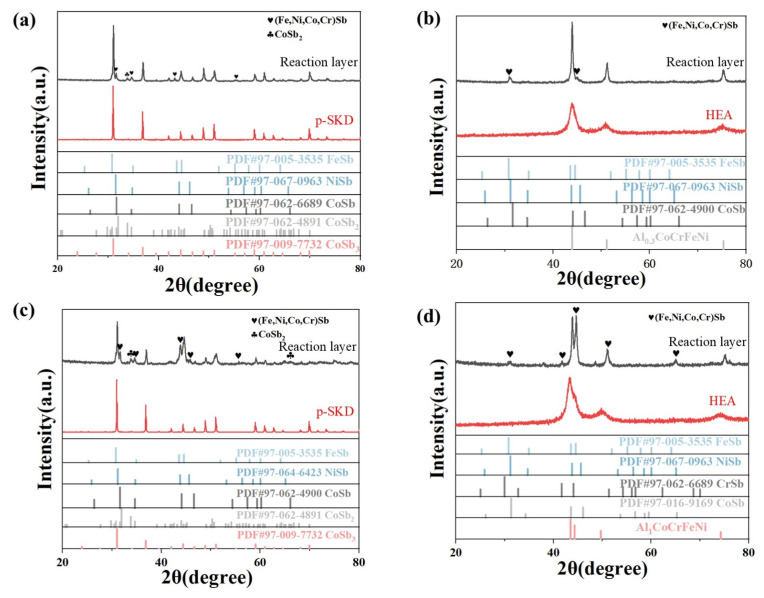
XRD patterns of interfacial reaction layers at Al_x_CoCrFeNi/p-SKD interfaces: (**a**,**b**) Al_0.3_CoCrFeNi/p-SKD interface, (**c**,**d**) Al_1_CoCrFeNi/p-SKD interface.

As shown in Figure 4a, the interfacial reaction layer formed at the skutterudite side of Al_0.3_CoCrFeNi exhibits diffraction peaks corresponding to a single predominant phase (apart from the skutterudite peaks). This phase possesses a hexagonal structure identical to NiSb, FeSb, and CoSb, with lattice parameters intermediate between these binary compounds. The EDS analysis suggests a possible (Fe,Ni,Co,Cr)Sb composition, indicating that the reaction layer consists of a multicomponent alloyed intermetallic phase with a hexagonal lattice structure. As shown in Figure 4b, the interfacial reaction layer on the high-entropy alloy side similarly demonstrates that no additional phases formed beyond the (Fe,Ni,Co,Cr)Sb intermetallic compound and the Al_0.3_CoCrFeNi high-entropy solid solution phase. This observation is corroborated by the EDS compositional analysis presented in Table 2, which confirms that the reaction layer on this side consists exclusively of a mixture of these two phases. The interfacial reaction layers formed by Al_1_CoCrFeNi show similar phase characteristics to those of Al_0.3_CoCrFeNi, where the skutterudite-side interface is dominated by a hexagonal (Fe,Ni,Co,Cr)Sb intermetallic phase, while the high-entropy alloy side consists of a dual-phase mixture of (Fe,Ni,Co,Cr)Sb and FCC+BCC solid solution.

Notably, the interfacial reaction layer formed on the high-entropy alloy side demonstrates a comparable morphology and phase composition to the original high-entropy alloy, whereas the skutterudite-side reaction layer exhibits distinct differences from the parent skutterudite material (Figure 3). This significant dissimilarity in the skutterudite-side interfacial structure is expected to predominantly influence the interfacial thermomechanical properties. Therefore, we focus our investigation on the growth behavior of the skutterudite-side reaction layer during thermal aging. Due to the non-uniform thickness distribution of the interfacial reaction layer, we performed statistical averaging of thickness measurements taken from at least five different locations. For the Al_0.3_CoCrFeNi/p-SKD interface, the reaction layer thickness increased from 2.5 μm (as-bonded) to 9.5 μm (after 16-day aging) and further to 14.2 μm (23-day aging). Correspondingly, the Al_1_CoCrFeNi /p-SKD interface showed thickness growth from 4.3 μm (initial) to 9.8 μm (16 days) and 12.3 μm (23 days). Compared with the Al_1_CoCrFeNi alloy, the Al_0.3_CoCrFeNi/p-SKD interfacial reaction layer exhibits slightly faster growth kinetics. Remarkably, both high-entropy alloys form defect-free interfaces after aging, with no observable diffusion voids or microcracks at the reaction zones. This indicates that the interfacial reaction layer exhibits favorable physicochemical compatibility with both skutterudite and high-entropy alloys, demonstrating balanced atomic interdiffusion across the interface. 

We statistically analyzed the thickness of the (Fe,Ni,Co,Cr)Sb intermetallic reaction layers at the interfaces of the two high-entropy alloy joints after aging at 723 K for different durations, as shown in Figure 3i,j. The growth of the interfacial reaction layer followed the square root time law (*Y* = *Y*_0_ + (*Dt*)^0.5^, where *t* is the aging time, *Y* is the thickness of the (Fe,Ni,Co,Cr)Sb layer at time *t*, *Y*_0_ is the initial thickness of the interfacial layer, and *D* is the growth rate as a function of temperature). The linear correlation coefficients (*R^2^*) of the fitted curves were all greater than 0.9, confirming that the growth of the interfacial layers is diffusion-controlled under the studied aging conditions. The extracted growth rates for the interfacial layers were 0.569 × 10^−16^ m^2^/s for Al_0.3_CoCrFeNi and 0.295 × 10^−16^ m^2^/s for Al_1_CoCrFeNi, both relatively low values, indicating that Al_x_CoCrFeNi serves as an effective diffusion barrier for elemental transport.

**Figure 5:**
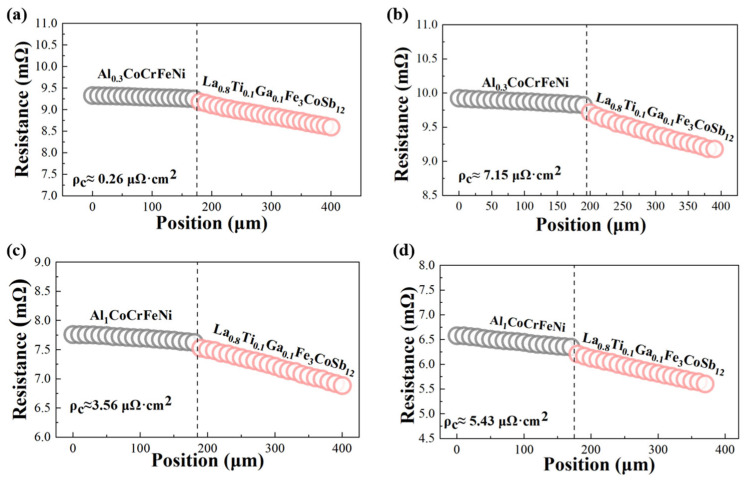
Contact resistivity of Al_x_CoCrFeNi/p-SKD interfaces before and after aging: (**a**) x = 0.3, as-bonded; (**b**) x = 0.3, aged 16 days; (**c**) x = 1, as-bonded; (**d**) x = 1, aged 16 days.

The low interfacial contact resistance between the reaction layer and thermoelectric materials is one of the critical factors ensuring high energy conversion efficiency in thermoelectric devices. Therefore, we employed the four-point probe method to characterize interfacial resistance. As shown in Figure 5, both high-entropy alloys exhibit ultralow interfacial resistivity. 

Pre-aging, Al_0.3_CoCrFeNi exhibited an interfacial resistivity of only 0.26 μΩ·cm^2^, which is significantly lower than Al_1_CoCrFeNi’s 3.56 μΩ·cm^2^. This is attributed to the thinner interfacial reaction layer formed in Al_0.3_CoCrFeNi after bonding. Following 16-day aging at 773 K, the interfacial resistivities of both high-entropy alloys increased with reaction layer thickening, converging to comparable values of 7.15 μΩ·cm^2^ for Al_0.3_CoCrFeNi and 5.43 μΩ·cm^2^ for Al_1_CoCrFeNi, both remaining below 10 μΩ·cm^2^. This demonstrates excellent thermal stability of interfacial contact resistance in both alloys. We also measured the electrical conductivity of Al_x_CoCrFeNi high-entropy alloys using the standard four-probe method. The FCC-structured Al_0.3_CoCrFeNi exhibited a conductivity of 2.95 × 10^5^ S/m, whereas the FCC+BCC-structured Al_1_CoCrFeNi showed a slightly higher value of 4.37 × 10^5^ S/m. This variation in electrical conductivity can be attributed to differences in the electronic transport properties induced by the change in crystal structure. Specifically, structural variations affect the electron relaxation time (*τ*), leading to different degrees of electron scattering. According to previous reports [36,37], the calculated *τ* values for BCC structures are generally higher than those for FCC structures, indicating reduced electron scattering in the BCC phase. These findings are consistent with our experimental observation that the electrical conductivity of Al_x_CoCrFeNi alloys varies with crystal structure.

**Figure 6:**
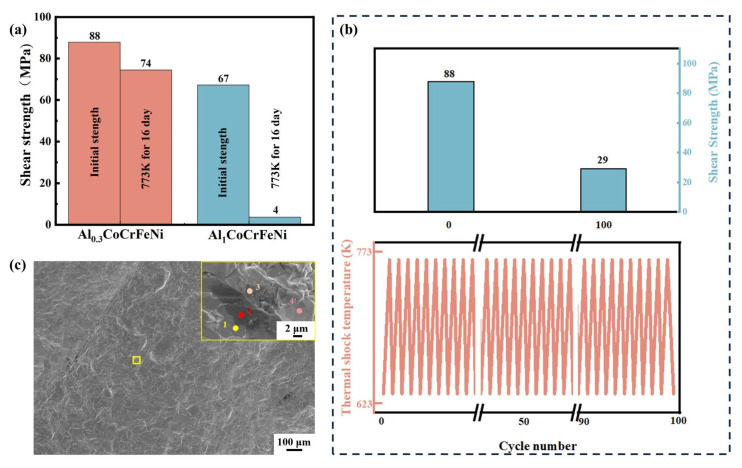
Mechanical properties of Cu-W/Al_x_CoCrFeNi/p-SKD joints: (**a**) shear strength before and after aging treatment, (**b**) shear strength after thermal cycling tests, and (**c**) fracture surface morphology of Cu-W/Al_0.3_CoCrFeNi/p-SKD joint.

To evaluate the mechanical properties and thermal stability of the high-entropy interfaces, shear strength tests were conducted on Cu-W/Al_x_CoCrFeNi/p-SKD joints. As shown in Figure 6a, both Cu-W/Al_0.3_CoCrFeNi/p-SKD and Cu-W/Al_1_CoCrFeNi/p-SKD joints exhibited excellent initial shear strengths of 88 MPa and 67 MPa, respectively. After aging at 773 K for 16 days, the Al_0.3_CoCrFeNi joint retained 74 MPa shear strength, representing only a 16% reduction, which ranks among the highest values reported in the current literature. In contrast, the Al_1_CoCrFeNi joint showed a dramatic decrease to merely 4 MPa after aging, essentially losing all load-bearing capacity. This remarkable difference likely originates from the distinct crystal structures of the two high-entropy alloys. Unlike the FCC+BCC dual-phase structure of Al_1_CoCrFeNi, the Al_0.3_CoCrFeNi alloy maintains a single-phase FCC solid solution structure, providing a superior plastic deformation capability [33,34,35]. This facilitates stress relief through localized interfacial deformation, thereby enhancing mechanical stability.

To evaluate the performance of high-entropy alloy interfaces under unstable thermal conditions, we further examined the mechanical stability of joints under extreme thermal shock. The Cu-W/Al_0.3_CoCrFeNi/p-SKD joints underwent 100 thermal cycles between 623 K and 773 K with heating/cooling rates of 100 K/min, holding for 5 s at both temperatures. As shown in Figure 6b, after 100 cycles, the shear strength of Al_0.3_CoCrFeNi joints decreased from 88 MPa to 29 MPa, demonstrating that extreme thermal shock conditions degrade interfacial mechanical properties more severely than isothermal aging, posing greater challenges to interfacial robustness. Nevertheless, the Cu-W/Al_0.3_CoCrFeNi/p-SKD joints maintained considerable strength (29 MPa) after 100 cycles, confirming Al_0.3_CoCrFeNi’s capability to ensure reliable thermomechanical stability between Cu-W electrodes and p-SKD thermoelectric materials.

As shown in Figure 6c, fracture surface analysis of the sheared Al_0.3_CoCrFeNi interface reveals a rugged morphology, indicating progressive crack deflection and propagation rather than abrupt brittle fracture. This fracture behavior demonstrates enhanced energy absorption during failure. Furthermore, EDS analysis (Table 3) shows that the fracture surface composition closely matches that of p-SKD, confirming that fracturing occurred within the skutterudite phase. This result further confirms that the p-SKD /Al_0.3_CoCrFeNi interface maintains superior bonding strength and does not constitute the structurally weakest region in the joint.

## 4. Conclusions

In summary, we propose the use of Al_x_CoCrFeNi high-entropy alloys as barrier layers to successfully achieve p-type skutterudite thermoelectric material/Cu-W electrode joints with both high electrical conductivity and superior mechanical properties. Notably, the Al_0.3_CoCrFeNi interface demonstrates exceptional performance with an ultra-low initial contact resistivity of 0.26 μΩ·cm^2^ and a remarkable shear strength of 88 MPa, representing one of the best reported combinations for thermoelectric material/metal joints. After aging at 773 K for 16 days, the interface maintains excellent stability with contact resistivity below 10 μΩ·cm^2^ and only 16% degradation in shear strength. Owing to the outstanding plastic deformation capability of the Al_0.3_CoCrFeNi’s FCC single-phase structure, the joint retains 29 MPa shear strength after 100 continuous extreme thermal cycles (623–773 K), demonstrating exceptional thermomechanical stability. These results establish Al_x_CoCrFeNi high-entropy alloys as ideal interfacial materials for realizing highly efficient and reliable thermoelectric devices.

## Figures and Tables

**Table 1:** Comparison of nominal and actual compositions (at.%) of Al_x_CoCrFeNi (x = 0.3, 1).

HEA		Al	Co	Cr	Fe	Ni
Al_0.3_CoCrFeNi	nominal compositions	7.2	23.2	23.2	23.2	23.2
	actual compositions	8.27	24.26	22.11	22.43	22.93
Al_1_CoCrFeNi	nominal compositions	20	20	20	20	20
	actual compositions	21.40	20.49	19.57	19.52	19.02

**Table 2:** EDS analysis results (at.%) for selected regions marked in Figure 2.

Point	Al	Ti	Cr	Fe	Co	Ni	Ga	Sb	La
A	0.04	0	2.08	23.30	11.87	14.31	0	50.01	0.34
B	0.02	0.32	2.16	25.13	9.29	13.22	0	50.19	0.07
C	20.54	0.01	19.55	22.34	18.68	13.59	0.34	3.83	0.02
D	1.1	0.06	20.13	11.81	15.57	16.04	2.26	33.02	0.06
E	25.28	0	28.85	13.65	17.22	12.34	0.51	2.12	0.03

**Table 3:** EDS analysis results of fracture surfaces.

Point	Al	Ti	Cr	Fe	Co	Ni	Ga	Sb	La
1	0.55	0.00	0.06	16.08	5.10	0.21	0.45	75.39	2.16
2	0.50	0.00	0.10	17.61	4.72	0.09	0.08	74.52	2.37
3	0.33	0.00	0.07	17.64	5.37	0.32	0.30	74.09	1.87
4	0.38	0.00	0.13	16.73	6.39	0.12	0.03	74.33	1.88
p-SKD	-	0.59	-	17.65	5.88	-	0.59	70.59	4.71

## Data Availability

The original contributions presented in the study are included in the article; further inquiries can be directed to the corresponding authors.
